# Supplementary material for: Development of a Macrophage-Related Risk Model for Metastatic Melanoma
Source: Int J Mol Sci. 2023 Sep 6;24(18):13752. doi: 10.3390/ijms241813752 (PMC10530689; doi:10.3390/ijms241813752)
Supplement: Supplementary file 1 [file ijms-24-13752-s001.zip › ijms-2556976-supplementary.pdf]

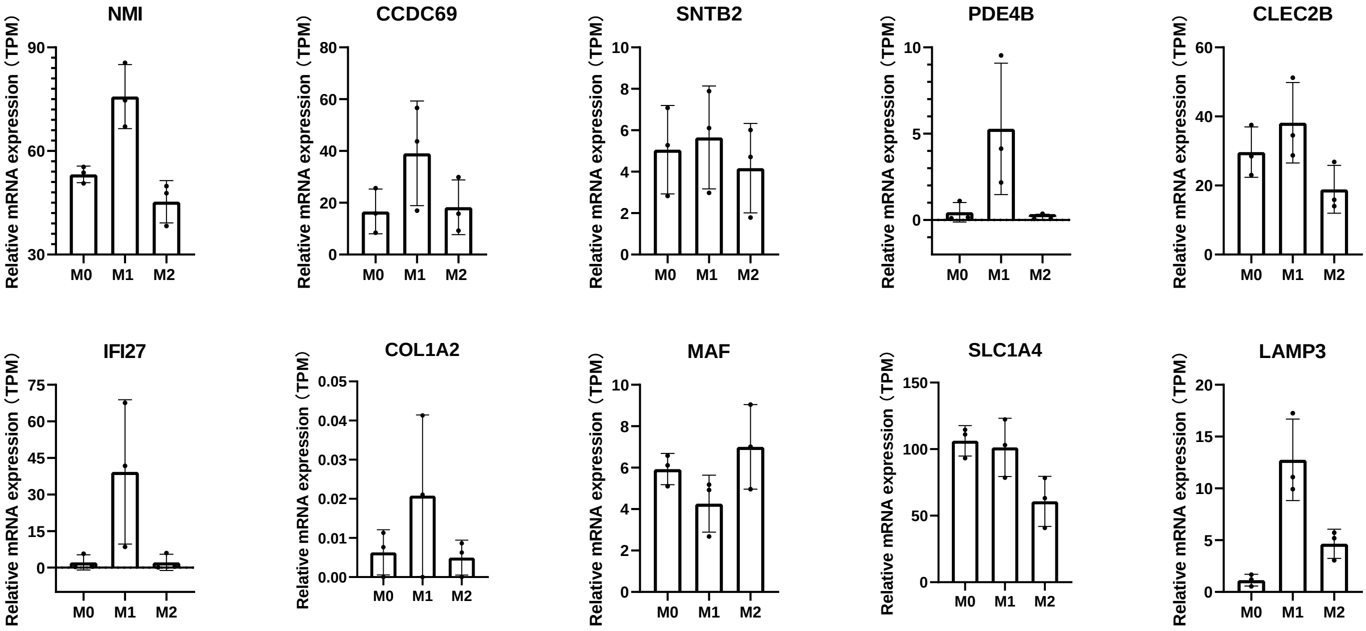

Supplementary figure 1. Expression of 10 genes in macrophages. Data were obtained from GSE195440, M0 , M1, M2 represent M0-type, M1-type, M2-type macrophages. Each group contains three samples.
